# Bright UV-C Phosphors with Excellent Thermal Stability—Y_1−*x*_Sc*_x_*PO_4_ Solid Solutions

**DOI:** 10.3390/ma15196844

**Published:** 2022-10-02

**Authors:** Dmitry Spassky, Andrey Vasil’ev, Vitali Nagirnyi, Irina Kudryavtseva, Dina Deyneko, Ivan Nikiforov, Ildar Kondratyev, Boris Zadneprovski

**Affiliations:** 1 Skobeltsyn Institute of Nuclear Physics, Lomonosov Moscow State University, Leninskiye Gory 1-2, 119991 Moscow, Russia; 2 Institute of Physics, University of Tartu, W. Ostwald Str. 1, 50411 Tartu, Estonia; 3 Department of Chemistry, Lomonosov Moscow State University, Leninskiye Gory 1-3, 119991 Moscow, Russia; 4 Laboratory of Arctic Mineralogy and Material Sciences, Kola Science Centre, Russian Academy of Sciences, 14 Fersman Street, 184209 Apatity, Russia; 5 Physics Department, Lomonosov Moscow State University, Leninskiye Gory 1-2, 119991 Moscow, Russia; 6 All-Russian Research Institute for Synthesis of Materials, Institutskaya Street 1, 601600 Alexandrov, Russia

**Keywords:** YPO_4_, ScPO_4_, solid solution, UV-C phosphor, energy transfer, bandgap, luminescence thermal stability, self-trapped exciton luminescence

## Abstract

The structural and luminescence properties of undoped Y_1−*x*_Sc*_x_*PO_4_ solid solutions have been studied. An intense thermally stable emission with fast decay (τ_1/e_ ~ 10^−7^ s) and a band position varying from 5.21 to 5.94 eV depending on the Sc/Y ratio is detected and ascribed to the 2p O-3d Sc self-trapped excitons. The quantum yield of the UV-C emission, also depending on the Sc/Y ratio, reaches 34% for the solid solution with *x* = 0.5 at 300 K. It is shown by a combined analysis of theoretical and experimental data that the formation of Sc clusters occurs in the solid solutions studied. The clusters facilitate the creation of energy wells at the conduction band bottom, which enables deep localization of electronic excitations and the creation of luminescence centers characterized by high quantum yield and thermal stability of the UV-C emission.

## 1. Introduction

Compounds with bright luminescence in the UV spectral region attract attention due to their application in photocatalysis, photochemistry and medicine, for disinfection, as persistent phosphors and scintillating detectors [1,2,3,4,5]. Emission in the UV spectral region is an advantageous feature for application in scintillators because it corresponds to the area where solar blind light detectors can be used in the open mode without shielding from daylight radiation [2]. Persistent UV emission can be applied in solar cells, photodynamic therapy and photo-catalysis [3]. UV phosphors with the emission in the UV-C range (200–280 nm) are of particular interest for medical applications. Their excellent bactericide properties are determined by high spectral overlap of the UV-C range and the germicidal effectiveness curve [1]. Moreover, the UV-C irradiation is a useful tool for killing superficial cancer cells without damage to deep tissue [6,7].

An intense emission in the UV-C spectral range is usually observed for wide-bandgap (E_g_ > 7 eV) phosphors doped with Ce^3+^, Pr^3+^ or Gd^3+^ rare-earth elements (REE) as well as s^2^ ions such as Pb^2+^ and Bi^3+^ [8,9,10,11,12,13,14]. The energy position of the non-shielded excited 5d state in Ce^3+^ and Pr^3+^ depends on the crystal field strength of the host material that allows to widely vary spectral position of the emission band. In particular, phosphates are attractive for the development of UV phosphors due to their large bandgap, high chemical stability and low synthesis temperature. However, for REE-doped compounds, a small Stokes shift of the emission results in strong emission reabsorption and phosphor degradation in UV emitting discharge lamps [15].

Compounds with intrinsic emission are also in the scope of interest for the UV phosphor applications. The luminescence of this type suffers from quenching processes at room temperature in the majority of compounds. However, an intense intrinsic emission in the UV-C region was reported for the BaZr_0.8_Hf_0.2_Si_3_O_9_ silicate solid solution, which demonstrated long term stability of its luminous flux better than that of the YPO_4_:Bi standard [16]. An intense UV emission has also been reported for ZnAl_2_O_4_ and attributed to oxygen vacancies [17,18] and excitons [19].

Solid solutions allow tailoring of phosphor properties for specific demands. Partial substitution of Al^3+^ atoms with Ga^3+^ in garnets allowed to suppress the adverse influence of electronic states of structural defects on energy transfer processes by enveloping the defect states within the conduction band [20,21]. The increase of light output as well as the enhancement of luminescence thermal stability has been observed for both activator and intrinsic emissions in different solid solutions [22,23,24,25,26,27]. The fluctuations of the energies of the electronic states forming the conduction band bottom and valence band top, introduced by the electronic states of substitutional atoms, promote localization of charge carries, thus constraining their migration in the crystal. The mean distance between electrons and holes decreases in such solid solution, thus increasing the probability of their radiative relaxation.

Based on the available knowledge, band structure modulation followed by charge carrier localization could be expected also in solid solutions of scandium and yttrium phosphates. Both phosphates are isostructural and characterized by wide bandgaps, while the difference between their bandgap values is rather big. The energies of exciton creation in ScPO_4_ and YPO_4_ were previously reported as E_ex_ = 7.45 and 8.55 eV, respectively [28]. Earlier, luminescence studies for the Y_1−*x*_Sc*_x_*PO_4_:Eu^3+^ solid solutions were performed in [29]. Alongside with the Eu^3+^ emission, an excitonic emission peaking around 250 nm has been observed in this compound at 8 K. It has been shown that the intensities of excitonic and Eu^3+^ emissions depend on the Sc/Y ratio and demonstrate the highest values for intermediate *x* values.

Here, we extend the studies of luminescence properties to the undoped Y_1−*x*_Sc*_x_*PO_4_ solid solutions. The process of energy transfer to the intrinsic emission centers is studied now in its pure form, without the influence from a competitive RE centers. Thermal stability of the intrinsic emission and its quantum yield at room temperature, which are essential for potential phosphor application, are studied as well. We demonstrate that the intrinsic luminescence peaking in the UV-C spectral region and possessing high quantum yield and thermal stability can be obtained for Y_1−*x*_Sc*_x_*PO_4_.

## 2. Experimental Section

### 2.1. Synthesis of Samples

The series of phosphates Y_1−*x*_Sc*_x_*PO_4_ (*x* = 0, 0.01, 0.05, 0.1, 0.2, 0.4, 0.5, 0.6, 0.8, 1) were synthesized by standard solid-state method from stoichiometric amounts of Y_2_O_3_ (99.99%), Sc_2_O_3_ (99.99%) and NH_4_H_2_PO_4_ (99.99%), which were mixed in different proportions in an agate mortar. Stoichiometric mixtures were heated slowly up to 1723 K, kept at this temperature for 10 h and then annealed for 50 h in the air in an alundum crucible. The chosen temperature allowed the formation of continuous raw solid solutions according to [30].

### 2.2. Structural Characterization

Powder X-ray diffraction (PXRD) patterns were collected on a Thermo ARL X’TRA powder diffractometer (Bragg-Brentano geometry, Peltier-cooled CCD detector, CuKα radiation, λ = 1.5418 Å). PXRD data were collected at room temperature in the 2θ range between 5° and 65° with a step interval of 0.02°. The Le Bail decomposition method was applied to determine the lattice parameters using the JANA2006 software (ver. 25.10.2015, JANA Inc., Universal City, TX, USA) [31,32].

### 2.3. Luminescence Characterization

Photoluminescence (PL) and photoluminescence excitation (PLE) spectra were recorded using a specialized setup for spectroscopy in the VUV energy range. A Hamamatsu L11798 deuterium lamp was used as an excitation source. The excitation wavelength was selected using a McPherson 234/302 vacuum monochromator. Samples were placed into a vacuum closed-cycle custom-made optical helium cryostat from ARS. Temperature was changed in the 5–350 K range using a temperature controller LakeShore331. PL signal was registered using a Shamrock 303i (Andor Technology) monochromator equipped with a Hamamatsu H8259-02 photon counting head. Emission spectra were corrected on spectral sensitivity function. The spectra were converted from a nanometer to an energy scale by the multiplication of emission intensity by the factor λ^2^ in each spectral point for the correct fitting of the PL spectra with the Gaussian functions.

Absolute quantum yield was measured on a laboratory setup under hydrogen discharge lamp irradiation. The irradiation wavelengths 160 nm (7.75 eV) and 176 nm (7.05 eV) were selected using a VMR-2 vacuum monochromator. The latter value is close to the characteristic radiation of mercury-free Xe discharge lamps (174 nm), which can be used for the UV-C luminescence excitation. The PL spectra of the sample and sodium salicylate were measured in equal geometric conditions with all necessary corrections including the spectral function of the detector sensitivity. The quantum yield values for Y_1−*x*_Sc*_x_*PO_4_ solutions were obtained relatively to that of sodium salicylate. These values were further normalized on the absolute quantum yield of sodium salicylate, which was determined as QY = 64% at room temperature [33].

Emission decay curves were measured at the pulsed cathodoluminescence setup described in detail in [34]. An electron beam with a broad spectrum, E_max_∼120 keV, pulse FWHM 200 ps, and peak electron current 60 A/cm^2^ was used for excitation. The samples were placed into a custom-made closed-cycle helium cryostat from ARS, which allowed to perform measurements in the temperature region 5–350 K. A Hamamatsu R3809U-50 MCP-PMT was used to record decay curves in a pulsed current mode. Its output was digitized by a LeCroy SDA 760Zi-A oscilloscope (6 GHz, 40 Gs/s).

### 2.4. Numerical Simulation of the Density of States of Solid Solutions

The densities of states of solid solutions were simulated for a qualitative explanation of the excitation spectra. The approach is based on the calculation of the potential landscape of the conduction band bottom in a model crystal with the substitution disorder in Sc and Y cations, similar to that described in [35]. This approach was generalized to a lattice with several equivalent cation positions in the unit cell with *I*41/*amd* symmetry. A periodic supercell with a size of 10 × 10 × 10 elementary cells containing 2000 cationic positions was taken for a simulation. The densities of states were estimated from the fluctuations of the landscape using the method of averaging the model density of states of a three-dimensional crystal, considering the shift of the potential at different points. The simulations were performed for the cases of (i) the absence of the correlation in the cations positions and (ii) when the location of Sc ions close to each other is preferable. In the latter case we suppose that the energy of the Sc-Sc pair formation in two adjacent positions equals to U_Sc-Sc_ = 0.3 eV. It implies the increase of the probability of adjacent location of two Sc ions 30 times in comparison to their random distribution, if we suppose that the crystal growth temperature is 1000 K (i.e., the ratio U_Sc-Sc_/kT = 3.5).

### 2.5. SEM Investigation

SEM observations and elemental mapping analysis of the Y_1−*x*_Sc*_x_*PO_4_ (*x* = 0, 0.01, 0.05, 0.1, 0.2, 0.4, 0.5, 0.6, 0.8, 1) samples were performed using a Tescan VEGA3 (Tescan, Brno, Czech Republic) scanning electron microscope equipped with an Oxford Instruments X-Max 50 silicon drift energy-dispersive X-ray spectrometry (EDX) system with AZtec (Oxford Instruments NanoAnalysis, Besançon, France) and INCA software (JANA, Inc., Universal City, TX, USA, Base Product package). Samples were coated with a thin layer of carbon for the SEM examinations. SEM images were acquired using secondary electron and backscattered electron imaging techniques. The EDX analysis results were based on the Sc_K_, Y_L_, and P_K_ edge lines. The oxygen content was not quantified by EDX.

## 3. Results and Discussion

### 3.1. Crystal Structure

**Figure 1:**
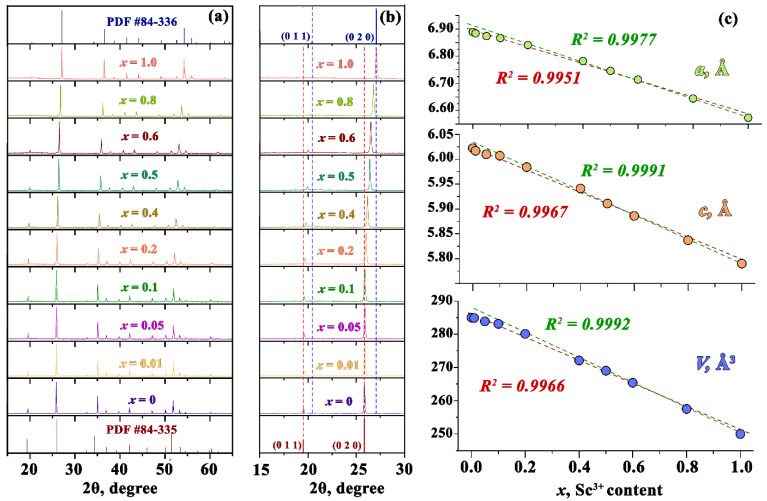
(**a**) PXRD patterns of Y_1−*x*_Sc*_x_*PO_4_ (*x* = 0, 0.01, 0.05, 0.1, 0.2, 0.4, 0.5, 0.6, 0.8, 1) and Bragg reflections of pure YPO_4_ (ICSD 84–335) and ScPO_4_ (ICSD 84–336). (**b**) The fragment of PXRD patterns of Y_1−*x*_Sc*_x_*PO_4_ (*x* = 0, 0.2, 0.4, 0.5, 0.6, 0.8, 1) in the region of selected XRD peak. (**c**) The dependence of the unit cell parameters *a* (**a**), *c* (**b**) and volume *V* (**c**) of the unit cell on Sc content for Y_1−*x*_Sc*_x_*PO_4_. The linear fit is done for the whole range of *x* values (red dashed curve) and for the *x* values in the range from 0.1 to 1 (green dashed curve).

All peaks in PXRD patterns for synthesized YPO_4_ correspond to PDF Card No. 84–335 while those for ScPO_4_—to PDF Card No. 84–336 (Figure 1a). No additional peaks have been found for the solid solutions, while the existing PXRD peaks shift gradually to higher 2θ values with the increase of *x* (Figure 1b). Thus, all synthesized samples are single-phased and crystallize in Xenotime-type structure with tetragonal syngony and space group *I*4_1_*/amd* (*D*^19^_4*h*_, No. 141, Z = 4). The metal atom is eight-coordinated to oxygen atoms with two different metal-oxygen distances, while phosphorus is four-coordinated forming a distorted PO_4_ tetrahedron in this structural type [36].

The calculated unit cell parameters are presented in Figure 1c and Table 1. The unit cell parameters decrease with *x* that is connected with the substitution of Y^3+^ (*r*_VIII_ = 1.02 Å) with smaller Sc^3+^ (*r*_VIII_ = 0.87 Å) [37] in the crystal structure. The dependence of unit cell parameters can be fitted with a linear function thus following Vegard’s law [38]. However, slight deviation from the linear dependence can be supposed for solutions with low Sc content. The deviation becomes more evident if the dependence is fitted by linear function for the solutions with Sc content from *x* = 0.1 to 1. Similar deviations were also observed in Gd(Nb_x_Ta_1−x_)O_4_ as well as some other solid solutions (see [39] and references therein). The deviations were attributed to inhomogeneous (in nanoscale) distribution of substitutional atoms.

### 3.2. SEM Analysis

**Figure 2:**
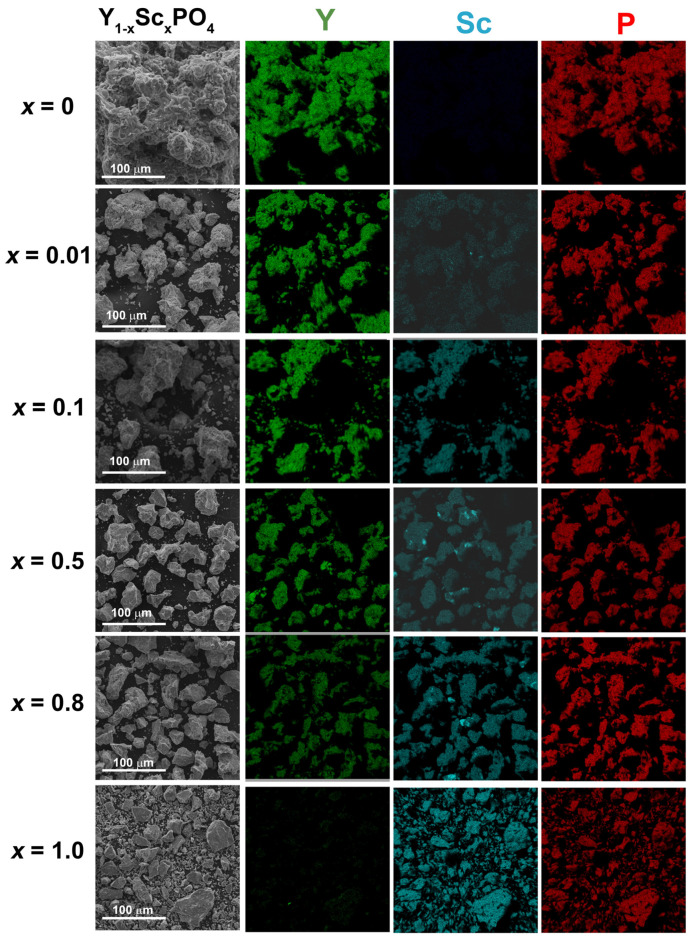
SEM images (left column) and elemental mapping of Y, Sc and P for Y_1−*x*_Sc*_x_*PO_4_ (*x* = 0, 0.01, 0.1 0.5, 0.8, 1.0).

SEM images of Y, Sc and P for Y_1−x_Sc_x_PO_4_ (x = 0, 0.01, 0.1 0.5, 0.8, 1.0) as well as elemental mapping of Sc, Y ad P atoms are presented in Figure 2. According to the obtained images, the synthesized powders consist of agglomerated particles with the size of few tens of microns. No clear dependence of particle size on Y/Sc ratio was found. The elemental mapping is also shown in Figure 2. Y, Sc and P elements are homogeneously distributed inside each particle. It is worth noting that the spatial resolution of the mapping does not allow to detect Sc clusters to be discussed below.

The calculated data on the Y:Sc:P ratio obtained by the EDX measurement are shown in Table 2. These data reveal that the obtained compositions are close to the expected ones. The results also demonstrate that the deviation of the dependence of lattice constants from the Vegard’s law (Figure 1) for low *x* values cannot be explained by inconsistency between the expected and real Y/Sc ratios in the samples.

### 3.3. Luminescence Properties of Y_1−x_Sc_x_PO_4_

**Figure 3:**
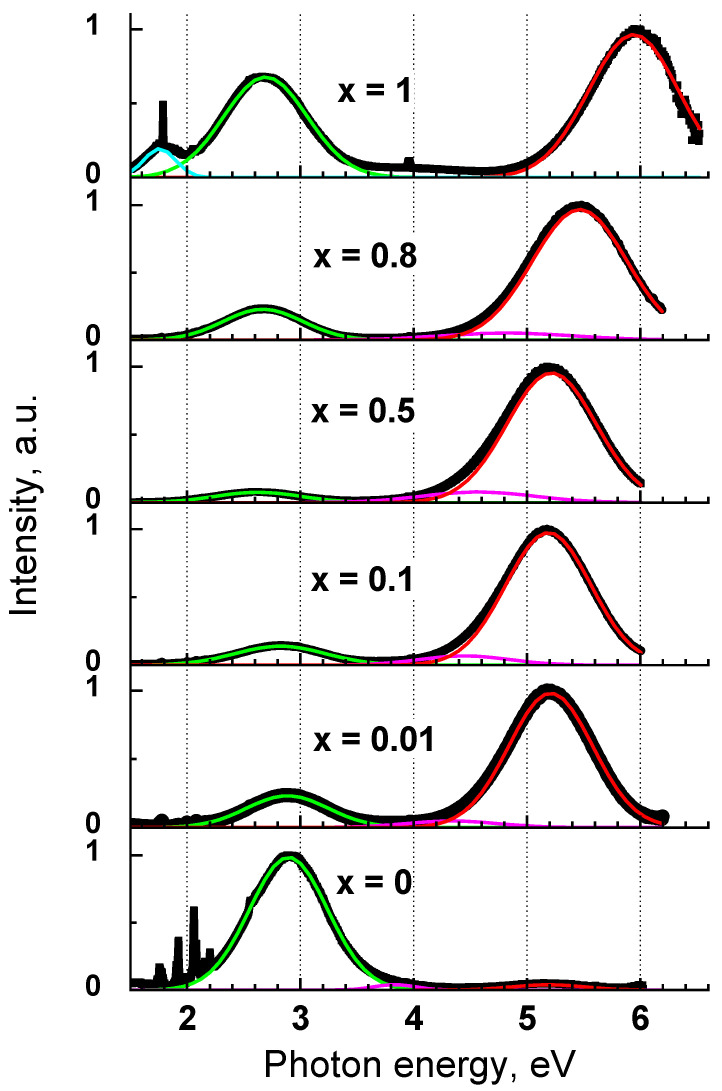
Photoluminescence spectra of Y_1−*x*_Sc*_x_*PO_4_ at E_ex_ = 7.75 eV (160 nm), T = 300 K. The Gaussian fit of the emission bands is presented with colored thin curves.

Photoluminescence spectra of selected Y_1−x_Sc_x_PO_4_ samples under the VUV excitation E_ex_ = 7.75 eV (λ_ex_ = 160 nm) are presented in Figure 3. All Sc containing phosphates demonstrate an intense emission band in the UV-C spectral region, while it is very weak in pure YPO_4_. The UV band is dominant in the spectrum of ScPO_4_ as well as solid solutions, even for the sample Y_0.99_Sc_0.01_PO_4_ with the lowest Sc concentration. The band maximum shifts from 5.94 eV (205 nm) to 5.2 eV (238 nm) with the change of x from 1 to 0.6, while its position does not change for lower Sc concentrations. Additional emission bands were also found in a longer-wavelength region. A very weak emission band peaking at 4.34–4.83 eV is observed in the UV spectral region and another emission band appears in the blue spectral region at 2.65–2.9 eV. The latter band dominates in the PL spectrum of YPO_4_ but it is weaker than the UV-C band in solid solutions. Only ScPO_4_ reveals a weak broad emission band in the red spectral region previously reported in [40]. The parameters of the emission bands obtained from the Gaussian fit of the emission spectra are presented in Table 3.

**Figure 4:**
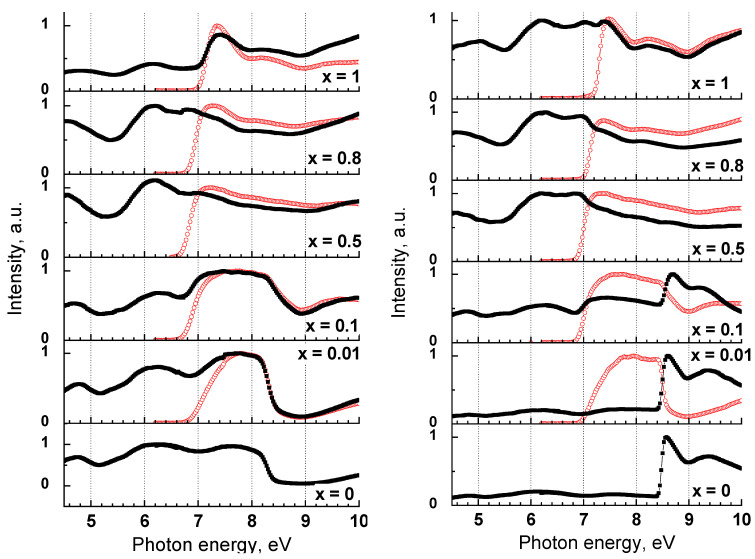
Excitation spectra of Y_1−*x*_Sc*_x_*PO_4_ for the UV-C emission (red circles) and defect-related emission (black squares) at 300 (**left panel**) and 7 K (**right panel**).

Photoluminescence excitation spectra of selected Y_1−x_Sc_x_PO_4_ samples are presented in Figure 4. In ScPO_4_, the first excitation peak is observed at the photon energy 7.5 eV corresponding to the energy of exciton creation [28]. The position of the low energy onset of the UV-C band excitation spectrum coincides with the edge of exciton creation and does not considerably change with *x*. The profile of the excitation spectrum changes upon the decrease of *x*. A pronounced excitation band in the region 7.0–8.5 eV becomes evident only in solid solutions with the low Sc content (*x* = 0.01, 0.1). Moreover, the intensity rise at the low-energy onset of the band is less steep for the sample with low Sc content. In the excitation spectra of the blue emission found at 2.7–2.9 eV, several broad bands peaking at ~4.8 and ~6.2 eV are observed in the transparency region of the crystal. Thus, the blue emission band can be related to the crystal structure defects. Previously, it was reported that the appearance of the blue band in ScPO_4_ depends on synthesis conditions [41] that confirms the conclusion. A sharp peak at 8.55 eV at T = 7 K corresponds to the exciton creation energy in YPO_4_. The corresponding exciton peak is also observed in the excitation spectra of the blue emission in solid solutions with low Sc concentration (*x* < 0.2), while it disappears for higher Sc concentrations.

**Figure 5:**
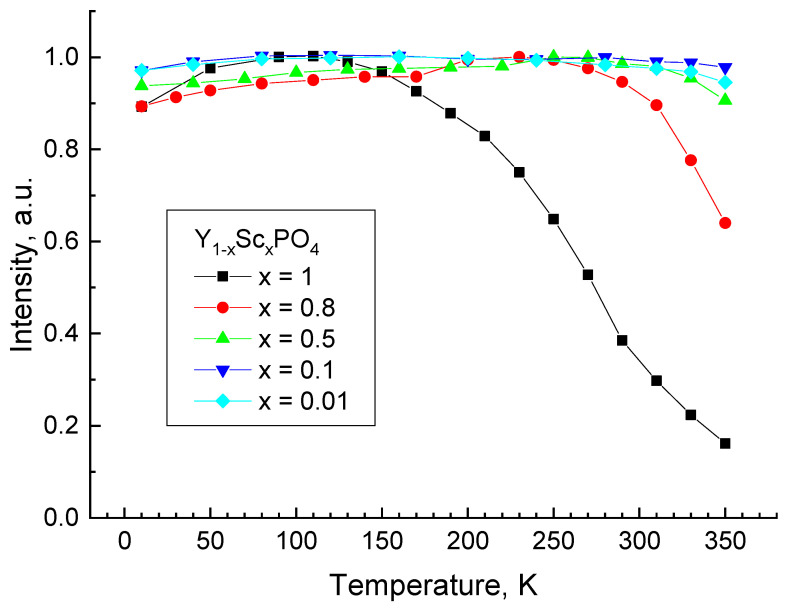
Temperature dependence of the UV-C band emission intensity in Y_1−*x*_Sc*_x_*PO_4_ at E_ex_ = 7.75 eV (λ_ex_ = 160 nm). The points were obtained by integrating of the PL signal in the wavelength region 190–265 nm (*x* = 1), 190–290 nm (*x* = 0.8) and 190–310 nm (*x* = 0.5, 0.1 and 0.01).

The temperature dependence of the UV-C band intensity is presented in Figure 5. The excitation energy E_ex_ = 7.75 eV (λ_ex_ = 160 nm) corresponds to the fundamental absorption region of Y_1−*x*_Sc*_x_*PO_4_ solid solutions with high Sc content or to the center of the broad excitation band of the UV-C emission for the samples with low Sc content. The quenching of the emission starts at T > 130 K for ScPO_4_, whereas the intensity is reduced by three times with temperature increase up to 300 K. The thermal stability of UV-C emission increases with the decrease of Sc content in solid solutions. The quenching starts at T > 290 K for *x* = 0.8, T > 330 K for *x* = 0.5, while it is stable up to the high-temperature limit of the performed experiment (350 K) for lower Sc concentrations.

### 3.4. Quantum Yield of Y_1−x_Sc_x_PO_4_

**Figure 6:**
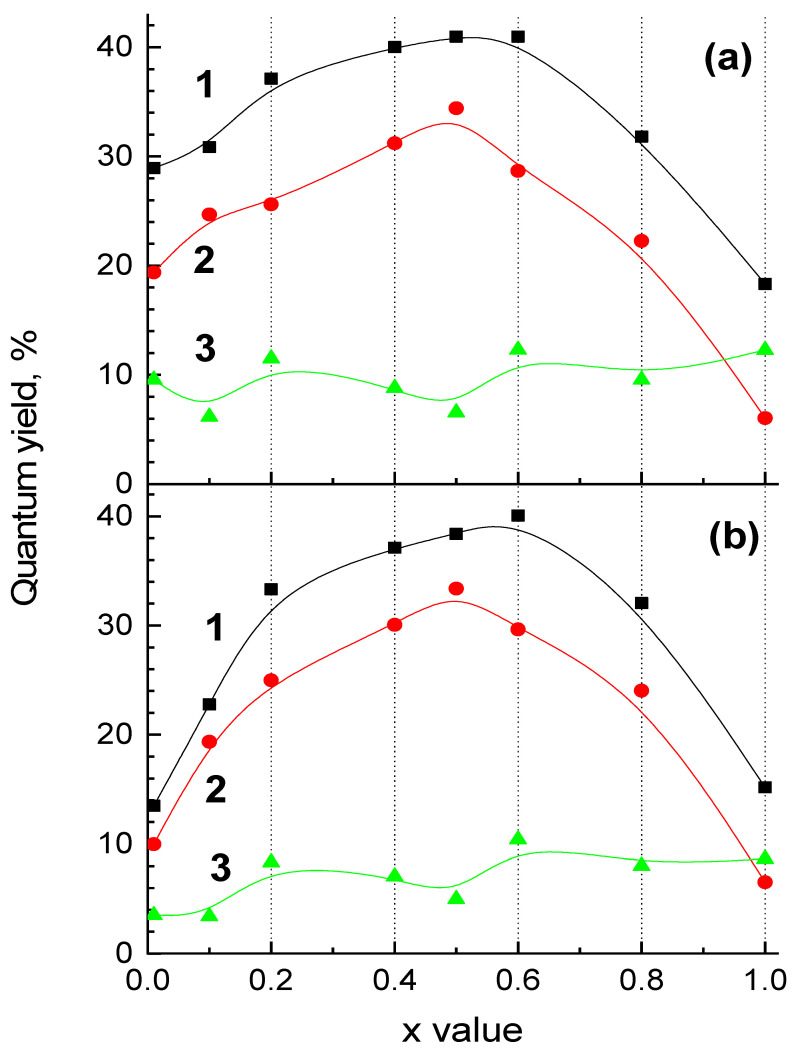
Quantum yield of Y_1−*x*_Sc*_x_*PO_4_ solid solutions at E_ex_ = 7.75 eV (λ_ex_ = 160 nm) (**a**) and E_ex_ = 7.05 eV (λ_ex_ = 176 nm) (**b**), T = 300 K. The signal was integrated over 200–700 nm spectral region (curve 1) as well as in the region of the UV-C (2) and blue (3) emission bands.

The quantum yield dependences on *x* value for the UV-C and blue emission bands are presented in Figure 6. The measurements were performed for excitation energies E_ex_ = 7.75 and 7.05 eV. The former value corresponds to the fundamental absorption region of solid solutions with *x* ≠ 0. The latter value corresponds to the region of fundamental absorption edge and is close to resonant transition in a Xe discharge lamp (174 nm). A clear dependence of the UV-C emission intensity on solid solution composition is observed. The quantum yield is maximal for the solution with equal concentrations of substitutional cations (*x* = 0.5), while it gradually decreases for higher and lower *x* values. A slight difference between these curves obtained at E_ex_ = 7.75 and 7.05 eV is observed for low *x* values, for which the quantum yield decrease is more pronounced for E_ex_ = 7.05 eV in accordance with a slight shift of the Urbach tail of the fundamental absorption edge to higher energies (Figure 4). The quantum yield of the UV-C emission for the brightest sample Sc_0.5_Y_0.5_PO_4_ was determined as 34%. In contrast to the UV-C emission, the defect-related blue emission band demonstrates no clear dependence on solid solution composition. The obtained values fluctuate in the region of 6–12% for E_ex_ = 7.7 eV and 3–10% for E_ex_ = 7.05 eV.

### 3.5. Cathodoluminescence Decay Characteristics of Y_1−x_Sc_x_PO_4_

**Figure 7:**
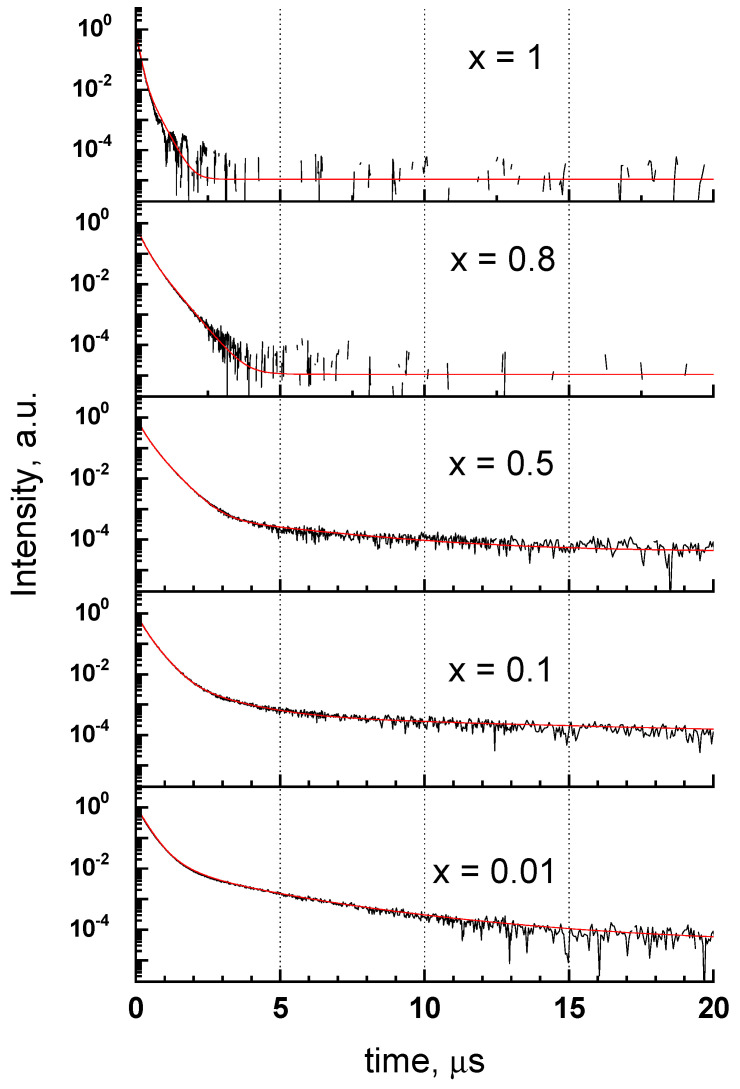
Decay curves of the UV-C (E_em_ = 5.2 eV for *x* = 0.01, 0.1, 0.5; 5.45 eV for *x* = 0.8 and 5.95 eV for *x* = 1) emission band of Y_1−*x*_Sc*_x_*PO_4_ at 300 K under pulsed electron beam excitation.

The decay curves of the UV-C emission band under excitation with pulsed electron beam are presented in Figure 7. The curves are not single exponential, and for the most part of solid solutions (*x* = 0.01–0.6) they could be fitted with the sum of four exponents describing the decayed part of the curve and an additional component related to the initial rise of the emission. The relatively slow rise component appears on top of prompt rise and its characteristic time varies from 3 to 10 ns without clear dependence on Sc content. The calculated decay times are presented in Table 4 alongside with simple τ_1/*e*_ values representing the time of the total emission intensity decrease by the factor of *e*. The main contribution into the decay of the UV-C emission (more than 90%) is made by the two relatively fast components with decay times of few hundreds of ns. The slowest component is characterized by decay times in a microsecond scale. The decay time of the slow component increases from 4.42 to 11.58 µs with the increase of Sc content from 0.01 to 0.2 and decreases down to 3.69 µs with further increase of Sc up to 0.6. This component cannot be observed in samples with higher Sc content. The decay acceleration for the UV-C emission in solid solutions with *x* = 0.8 and 1 is attributed to thermal quenching starting in these compounds below 290 K (Figure 6).

### 3.6. Discussion

#### 3.6.1. Origin of UV-C Emission Band

The UV-C emission band has been previously reported for ScPO_4_ [42,43,44]. The band was ascribed to the emission of self-trapped excitons (STE) with a hole component at the 2p O states. The localization of an electron component is a point of discussion. It was attributed to the 3dSc states supposing that scandium states form the bottom of the conduction band [43] or the exciton localization was related to the PO_4_ molecular ion considering the observation of a similar UV emission band in other phosphates such as AlPO_4_ and GaPO_4_ [44].

The contribution of Sc states to the formation of the conduction band bottom was predicted by band structure calculations [45,46]. Their contribution into the band structure of Y_1−*x*_Sc*_x_*PO_4_ in the vicinity of the bandgap can be also deduced from the excitation spectra presented in Figure 4. The excitation spectrum of the 2.9-eV defect-related emission in YPO_4_ consists of several excitation bands peaking at 4.8, 6.1 and 7.5 eV in the transparency region of the crystal and a sharp onset at E_ex_ > 8.4 eV (Figure 4b). The onset is related to the Urbach tail of the fundamental absorption, while the first peak at 8.55 eV localized above this edge is attributed to exciton formation in YPO_4_ [28] with the hole component originating from the 2p O states and electron component confined to the 4d Y states. This sharp peak is also observed in the excitation spectra of defect-related emission for solid solutions with low Sc content (*x* ≤ 0.2) indicating high efficiency of energy transfer from the 2p O-4d Y excitons to defect emission centers.

The fundamental absorption edge is shifted by ~1 eV to the low-energy region in ScPO_4_. The shift is clearly observed in the excitation spectrum of the UV-C emission, demonstrating the onset at the lower energies E_ex_ > 7.15 eV. The first excitation peak at 7.5 eV is related to the exciton creation and its position corresponds perfectly to the value previously reported in [28]. The bandgap decrease with the substitution of Y with Sc is related to the low-energy shift of the conduction band, which occurs due to the lower energies of the 3d Sc states. Therefore, we suppose that the conduction band bottom in Y_1−*x*_Sc*_x_*PO_4_ (*x* ≠ 0) is formed mainly by the 3d Sc states, whose energy is considerably (>1 eV) lower than that of the 4d Y states.

The role of Sc in the formation of emission centers has been previously revealed for various oxides doped with Sc. The Sc doping usually resulted in the appearance of an emission band in the UV spectral region. An additional emission band peaking at 5.6 eV appears in Al_2_O_3_ doped with Sc, whereas it is attributed to electron recombination with holes localized at oxygen ions near Sc^3+^ centers [47]. The spectra of Sc-doped LuAG demonstrate an emission band peaking in the region 265–290 nm, which originates from radiative recombination of an exciton situated near Sc^3+^ [48]. The decay characteristics of Sc emission in LuAG as well as in some other garnets have been studied in [49]. The emission decay time τ_1/e_ for this band was estimated as hundreds of ns that also corresponds to the values obtained for the UV-C emission bands in Y_1−*x*_Sc*_x_*PO_4_ (Table 4). Therefore, we conclude that the UV-C band in Y_1−*x*_Sc*_x_*PO_4_ (*x* ≠ 0) is of intrinsic origin and scandium electronic states play an essential role in the formation of the corresponding emission center. Namely, an electron component of a self-trapped exciton in Y_1−*x*_Sc*_x_*PO_4_ (*x* ≠ 0) is localized at the 3d Sc states. In such case, the presence of slow microsecond components observed in the decay of the UV-C emission is related to delayed recombination due to intermediate localization of charge carriers at shallow traps.

It is worth noting that a very weak UV-C band was also observed by us for YPO_4_ (Figure 3) similarly to that previously reported in [50]. Judging from its excitation spectra, this band most likely arises due to the contamination of YPO_4_ with the traces of Sc. Actually, even low controllable admixture of Sc into YPO_4_ (solid solution with x = 0.01) results in the appearance of the intense UV-C emission band, which dominates in the emission spectrum. It is worth noting that only scandium charge transfer excitons show radiation recombination, whereas yttrium exciton emission is not observed at all. This fact can be possibly connected with larger Stokes shift of yttrium exciton emission and non-radiative quenching of such excitons during the relaxation process due to the crossing of excited and ground levels of the configurational diagram of corresponding emission center.

#### 3.6.2. Influence of Solid Solution Clusterization on the Energy Transfer Processes in Y_1−x_Sc_x_PO_4_

The increase of quantum yield for the intermediate values of *x* is frequently observed for solid solutions [22,23,24]. The effect of luminescence intensity increase in solid solutions has been previously ascribed to the formation of clusters, i.e., the solution areas enriched with one kind of the substitutional cations. The clusters limit the mean path length of the separated geminate charge carriers, thus increasing the probability of their radiative recombination.

The presented experimental results may indicate the presence of clusters in the Y_1−x_Sc_x_PO_4_ solid solutions. The presence of clusters should lead to inhomogeneous band broadening in both the STE emission and luminescence excitation spectra. From Table 3, it follows that the FWHM of the STE emission is 0.8 eV for ScPO_4_ and 0.74 eV for the case of very low (uncontrolled) concentration of scandium impurity in YPO_4_, while it reaches up to 0.83 eV for intermediate concentrations of the substitutional ions. The width of the STE emission is determined by the sum of homogeneous and inhomogeneous broadenings. The main reason of the homogeneous broadening is the interaction of charge transfer exciton with phonons. The inhomogeneous broadening is due to the variation of exciton energies and electron-phonon interaction in different clusters. The data from Table 3 show that inhomogeneous broadening of the emission band w is about 0.1 eV. The broadening depends on cluster size and configuration. The obtained low value of w implies a weak overlap of the wave functions of two neighboring cations in phosphates.

The behavior of excitation spectra will be discussed further after the presentation of the results of numerical simulations, which will be used for their qualitative explanation. The numerical simulation of substitutional cations distribution, fluctuations of potential and densities of states have been performed for the cases of random distribution of substitutional cations and correlations in the distribution of Sc ions (formation of Sc clusters).

**Figure 8:**
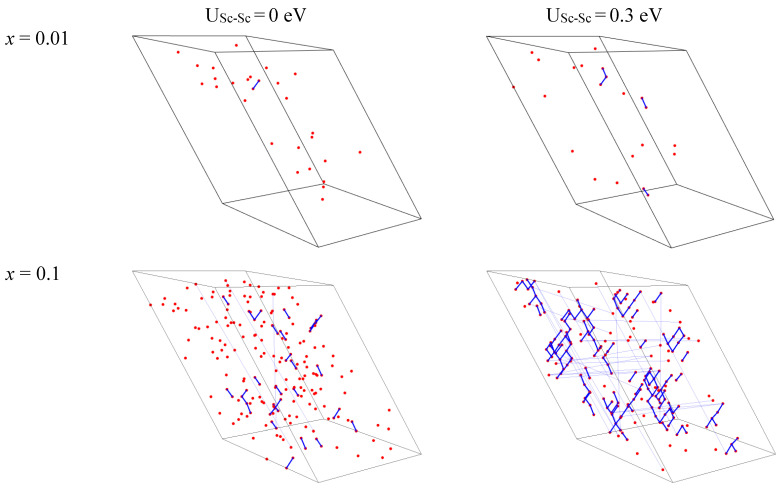
Examples of Sc ion location in the supercell without (left column) and with (right column) account of the correlations in ions location for two concentrations of Sc (*x* = 0.01 and 0.1). Sc ions are shown by red points while blue lines are connecting adjacent Sc ions. Thin blue lines are connecting adjacent Sc ions with account of supercell periodicity (Sc ions located at opposite sides of the supercell).

The distribution of Sc ions in the supercell for two Sc concentrations x = 0.01 and 0.1 for the cases of the absence and presence of Sc-Sc correlation are presented in Figure 8. In the case of the absence of correlation, the clusters consisting of two or more scandium ions are formed randomly. The probability of their creation is described by a law similar to Poisson’s one and there are about 20 Sc-Sc pairs for 200 Sc ions (*x* = 0.1 in the case of the 10 × 10 × 10 supercell) when the correlation is not considered. However, the formation of clusters consisting of several Sc ions is observed even for the lowest Sc concentration when the correlation is considered. Such clusters are not dense, the number of ions in a cluster increases as dα, where *α* ≈ 1.5 is the fractal dimension and d is a cluster size (maximal distance between two arbitrary Sc ions in the cluster). Please note that for case of 1D (linear) clusters (chains) *α* = 1, whereas for 3D dense spherical clusters *α* = 3. The values of d of some clusters can even exceed the linear sizes of the supercell. For U_Sc-Sc_ = 0.3 eV and *x* ≥ 0.2 the maximal value of d could increase to infinity which means that percolation appear (most of small clusters are combined into one giant cluster). For U_Sc-Sc_ = 0 eV such giant clusters appear only at *x* ≥ 0.5. We suppose that the possible deviation from the linear dependence of the unit cell volume on the scandium concentration, which are supposed for *x* < 0.2 (see Figure 1) can be associated with the appearance of such percolation chains of ions.

**Figure 9:**
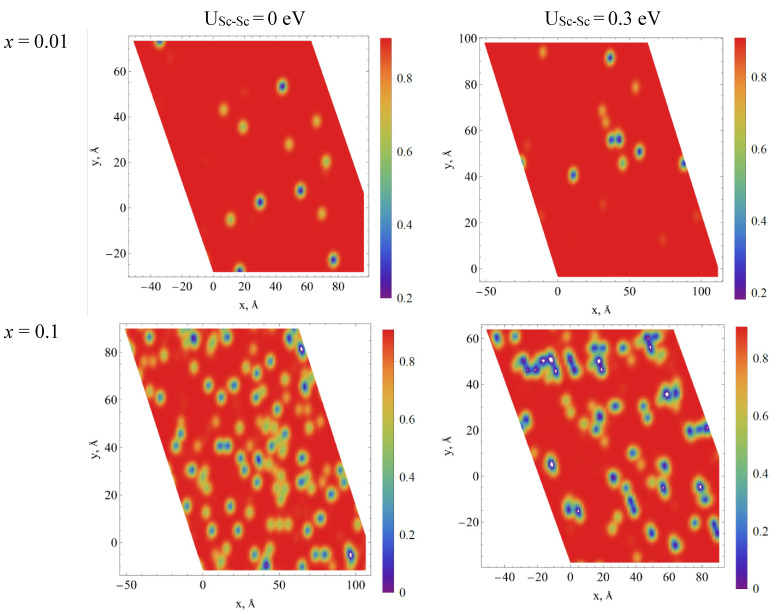
Examples of 2D cross-section of potential landscape without (**left column**) and with (**right column**) account for the Sc-Sc correlation for *x* = 0.01 and *x* = 0.1.

The difference between the pseudopotentials of yttrium and scandium ions was introduced for the construction of fluctuations of a potential at the conduction band bottom. The difference of the energies of the conduction band bottom in ScPO_4_ and YPO_4_ is determined as 1.5 eV. It is worth noting that the difference of the bandgap values of YPO_4_ and ScPO_4_ is smaller (~1.1 eV). The 1.5 eV difference between lower states of conduction band is partially compensated by the 0.4 eV high energy shift of the valence band top with the decrease of x (higher Y content). The corresponding shift of the valence band top has been predicted by calculations [28] and shown experimentally by the shift of the 2p O-4f Eu charge transfer excitation band in Y_1−x_Sc_x_PO_4_:Eu [51]. Considering the difference in the energies of the conduction band bottom, the difference in pseudopotential can be described as a Gaussian with an amplitude of 1.3 eV. The half-width of the Gaussian is chosen so that the pseudopotential of the yttrium ion at the location of an adjacent yttrium ion is 0.1 of its maximum. In this case, the average value of the potential in ScPO_4_ relative to YPO_4_ will be ~1.5 eV. A potential landscape was constructed using these parameters for solid solutions with different Sc concentrations. The examples of a 2D cross-section of potential landscape with and without account for the Sc-Sc correlation are presented in Figure 9.

As follows from Figure 9, a large difference between the energies of the conduction band bottom of ScPO_4_ and YPO_4_ results in the creation of deep energy wells. The energy wells at the bottom of the conduction band correspond to the local areas in the superlattice enriched with Sc and they are most pronounced in solutions with low Sc content (*x* < 0.2) for the case of Sc-Sc correlation. The creation of the energy wells will influence the relaxation of charge carriers at the stages of thermalization and migration of electrons via the conduction band states. In particular, it will prevent the separation of charge carriers due to migration and facilitate the creation of excitons. The localization of electrons at the bottom of the conduction band occurs at the 3d Sc states. This feature explains an unusually high quantum yield of the UV-C emission even for the lowest Sc concentrations because the localization of the electron components of self-trapped excitons takes place at these states. In the samples with higher Sc content (*x* > 0.2), the possibility of electron migration along the Sc chains restores again, causing additional energy losses. As a result, the quantum yield for the brightest sample with *x* = 0.5 is only two-fold higher than for the sample with the lowest Sc concentration (*x* = 0.01). The UV-C emission is also characterized by very good thermal stability. Its quenching starts at temperatures above 300 K for the most part of solid solutions (*x* < 0.8). It has been previously shown that thermal quenching of self-trapped excitons occurs due to their hopping diffusion in the crystal [52]. Excitons, whose localization is enhanced by the energy well creation will have lower probability of hopping diffusion or thermal disintegration, which would result in thermal quenching of luminescence.

**Figure 10:**
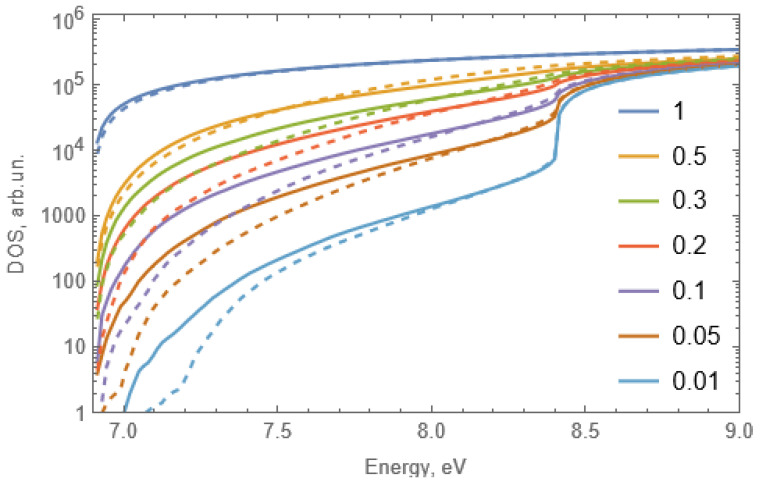
Density of states of a model crystal with different scandium concentrations (indicated in the legend) for the case when correlations in the arrangement of scandium ions are taken into account (solid curves) and in the absence of correlations (dashed curves). The density of states is estimated with the account for potential fluctuations.

The possibility of Sc-Sc correlation with the creation of clusters follows from the behavior of the excitation spectra of the UV-C emission, especially of their low energy onset. The density of states of solid solutions were calculated from the fluctuations of potential landscape for the case of random distributions of substitutional cations and Sc-Sc correlation. The results of simulation are presented in Figure 10. Since the absorption coefficient in the zero approximation will be proportional to the density of states, the normalization coefficient is chosen so that the numbers on the vertical axis correspond to the value of the absorption coefficient in cm^−1^. In particular, in the region of the fundamental absorption, the coefficient is about 10^5^–10^6^ cm^−1^.

The presented density of states can be used for the qualitative explanation of the excitation spectra. Excitation radiation will be completely absorbed in a sample with thickness ~1 mm if the absorption coefficient is higher than 10 cm^−1^. Thus, the edge of the excitation spectrum for a sample with *x* = 0.01 will be at 7.1 eV in case of the Sc-Sc correlation and at 7.25 eV in the case of the absence of correlation. The corresponding value decreases to 6.9 eV for higher concentrations regardless of the chosen model. The simulation predicts negligible shift of the excitation onset for Sc concentrations higher than 0.01 in case of the presence of Sc-Sc correlation while a gradual shift of the onset can be expected in the case of random Sc distribution.

Let us consider the modification of the excitation spectra with the change of Sc concentration (Figure 3). There is no gradual decrease of the bandgap energy with the increase of Sc content in solid solution. Instead, a slight shift of the onset to higher energies is observed with the increase of Sc content from 0.2 to 1 that is related to the shift of the valence band top [28,51]. Therefore, comparison with the experimental excitation spectra shows that a model that considers the Sc-Sc correlation and, consequently, the formation of clusters of scandium ions, is more plausible.

The electron transitions involving the 3d Sc states are clearly observed in the excitation spectra of the UV-C emission for solid solutions with relatively low Sc content (*x* ≤ 0.2). The UV-C emission is efficiently excited in the pronounced broad band in the region 7.0–8.3 eV. The excitation band is related to the 2p O-3d Sc transitions and represents the contribution of the 3d Sc states into the formation of the conduction band bottom. The increase of Sc concentration results in the broadening of the Sc excitation band and increase of the probability of energy transfer from yttrium excitations to scandium states. The latter effect is demonstrated by less prominent decrease of excitation efficiency for photon energies above 8.3 eV for *x* = 0.1 in comparison with *x* = 0.01. The migration of electrons over Sc subsystem results in the decrease of light yield when Sc clusters become infinite and electron with sufficient kinetic energy can migrate away from the hole. This effect becomes more pronounced for high concentration of Sc (*x* = 0.8 and *x* = 1) when the excitation spectrum shows a decrease at energies above 7.4 eV. At the same time, excitation spectra demonstrate no features in the region corresponding to yttrium exciton formation, since all electrons pass to scandium states during the relaxation. The features of excitation spectrum become rather sharp for *x* = 1, since in this case, the photon absorption occurs with corresponding transitions at Г point. According to band structure calculations for ScPO_4_ [46], the crystal has indirect bandgap and the energy at the Г point is 0.25 eV higher than the minimum of the conduction band. It is worth noting that the features in the excitation spectra become smooth in solid solutions. The smoothing is connected with the violation of the momentum conservation law in clusters, since their sizes are much less than the photon wavelength, thus resulting in the increase of the probability of indirect transitions.

## 4. Conclusions

Undoped Y_1−*x*_Sc*_x_*PO_4_ solid solutions were synthesized by solid-state technique and their structural and luminescence properties were evaluated. It is shown that all studied samples crystallize in a zircon-type structure forming a continuous set of solid solutions. The lattice parameters gradually decrease with the *x* value. An intense emission in the UV-C spectral region has been detected in the Y_1−*x*_Sc*_x_*PO_4_ solid solutions (*x* ≠ 0). Spectral position of the emission band depends on the Y/Sc ratio, changing from 5.94 eV at *x* = 1 to 5.21 eV at *x* ≤ 0.6. The emission is characterized by absolute quantum yield up to 34% for *x* = 0.5 at 300 K and decay time τ_1/e_ ~ 10^−7^ s. It is ascribed to the 2p O-3d Sc self-trapped excitons. The thermal stability of this UV-C emission increases with the decrease of the Sc content, whereas the threshold of thermal quenching exceeds 300 K for solutions with *x* < 0.8. Numerical simulation of substitutional cations spatial distribution describing the potential landscape at the conduction band bottom is performed with and without account of the correlated Sc-Sc pairs formation. It is shown by combined analysis of theoretical and experimental data that the case of Sc-Sc correlation is realized in Y_1−*x*_Sc*_x_*PO_4_ solid solutions. It results in the formation of Sc clusters, which facilitate the creation of energy wells at the conduction band bottom formed by the 3d Sc states even for low concentrations of Sc. It is concluded that clusters formation is preferable for deep localization of excitons, resulting in high thermal stability and quantum yield of the UV-C emission. The results of the studies demonstrate that Y_1−*x*_Sc*_x_*PO_4_ solid solutions can be considered as bright UV-C phosphors with relatively fast emission decay and excellent thermal stability in ambient conditions.

## Figures and Tables

**Table 1:** Lattice parameters of Y_1−*x*_Sc*_x_*PO_4_.

*X*	*a*, Å	*c*, Å	*V*, Å^3^
0	6.880(3)	6.019(5)	284.9(7)
0.01	6.879(1)	6.017(2)	284.8(1)
0.05	6.872(1)	6.010(4)	283.8(2)
0.1	6.867(2)	6.007(1)	283.2(5)
0.2	6.844(1)	5.987(3)	280.4(5)
0.4	6.794(3)	5.947(1)	274.4(7)
0.5	6.746(4)	5.911(1)	269.0(4)
0.6	6.714(3)	5.886(6)	265.4(1)
0.8	6.642(4)	5.836(5)	257.5(1)
1	6.571(4)	5.789(1)	249.9(3)

**Table 2:** EDX data for Y_1−*x*_Sc*_x_*PO_4_.

		Ratio	
*X*	Y	Sc	P
0	1.01 ± 0.03	0	1.00 ± 0.03
0.01	0.98 ± 0.03	0.01 ± 0.01	1.00 ± 0.02
0.05	0.93 ± 0.02	0.07 ± 0.04	1.00 ± 0.03
0.1	0.86 ± 0.04	0.08 ± 0.02	1.00 ± 0.03
0.2	0.76 ± 0.11	0.23 ± 0.11	1.00 ± 0.11
0.4	0.61 ± 0.09	0.35 ± 0.11	1.00 ± 0.06
0.5	0.48 ± 0.06	0.50 ± 0.08	1.00 ± 0.04
0.6	0.35 ± 0.02	0.63 ± 0.09	1.00 ± 0.08
0.8	0.18 ± 0.05	0.84 ± 0.04	1.00 ± 0.09
1.0	0	1.02 ± 0.03	1.00 ± 0.07

**Table 3:** Parameters of deconvolution of emission spectra into main Gauss components—peak position and FWHM (in brackets), eV.

x Value	UV-C Band	Defect 1	Defect 2
0	5.19 (0.74) *	3.82 (0.36)	2.89 (0.69)
0.01	5.21 (0.74)	4.34 (0.81)	2.88 (0.72)
0.1	5.19 (0.75)	4.44 (0.79)	2.82 (0.79)
0.5	5.21 (0.79)	4.57 (0.99)	2.62 (0.75)
0.8	5.46 (0.83)	4.83 (1.16)	2.67 (0.7)
1	5.94 (0.79)		2.69 (0.73)

* UV-C emission band in YPO_4_ is related to the uncontrollable Sc impurity in the sample.

**Table 4:** Decay components τ_dec_ and their weight (%) as well as τ_1/e_ at T = 290 K.

x Value	τ_dec_, ns	τ_1/e_, ns
0.01	254 (73%) 535 (18%) 1790 (6%) 4240 (3%)	294
0.05	134 (16%) 333 (77%) 1580 (6%) 7780 (1%)	259
0.1	166 (23%) 345 (68%) 1120 (7%) 11,370 (2%)	265
0.2	197 (42%) 378 (48%) 802 (8%) 11,580 (2%)	276
0.4	196 (53%) 433 (35%) 628 (11%) 6360 (1%)	256
0.5	34 (1%) 192 (42 %) 430 (56%) 3201 (1%)	253
0.6	32 (1%) 192 (44 %) 424 (54%) 3690 (1%)	250
0.8	26 (2%) 158 (44%) 357 (54%)	162
1	10 (4%) 92 (92%) 413 (4%)	56

## Data Availability

The data presented in this study are available on request from the corresponding author.
